# Seeing Emotion with Your Ears: Emotional Prosody Implicitly Guides Visual Attention to Faces

**DOI:** 10.1371/journal.pone.0030740

**Published:** 2012-01-30

**Authors:** Simon Rigoulot, Marc D. Pell

**Affiliations:** 1 McGill University, Faculty of Medicine, School of Communication Sciences and Disorders, Montreal, Quebec, Canada; 2 McGill Centre for Research on Brain, Language and Music, Montreal, Quebec, Canada; Federal University of Rio de Janeiro, Brazil

## Abstract

Interpersonal communication involves the processing of multimodal emotional cues, particularly facial expressions (visual modality) and emotional speech prosody (auditory modality) which can interact during information processing. Here, we investigated whether the implicit processing of emotional prosody systematically influences gaze behavior to facial expressions of emotion. We analyzed the eye movements of 31 participants as they scanned a visual array of four emotional faces portraying fear, anger, happiness, and neutrality, while listening to an emotionally-inflected pseudo-utterance (*Someone migged the pazing*) uttered in a congruent or incongruent tone. Participants heard the emotional utterance during the first 1250 milliseconds of a five-second visual array and then performed an immediate recall decision about the face they had just seen. The frequency and duration of first saccades and of total looks in three temporal windows ([0–1250 ms], [1250–2500 ms], [2500–5000 ms]) were analyzed according to the emotional content of faces and voices. Results showed that participants looked longer and more frequently at faces that matched the prosody in all three time windows (emotion congruency effect), although this effect was often emotion-specific (with greatest effects for fear). Effects of prosody on visual attention to faces persisted over time and could be detected long after the auditory information was no longer present. These data imply that emotional prosody is processed automatically during communication and that these cues play a critical role in how humans respond to related visual cues in the environment, such as facial expressions.

## Introduction

From an information processing standpoint, engaging in a “routine” conversation is rather complex; to understand a speaker's intentions listeners must carefully attend to and decipher cues encountered in different sensory modalities (vision, audition) and in several communication channels at once. In terms of the channels involved, listeners analyze the linguistic content of speech while interpreting the relational significance of vocal inflections in speech (i.e., speech *prosody*) and other extra-linguistic cues such as facial expressions and body movements. Given these different sources of social information that must be compared and integrated in some manner during interpersonal events, it is not surprising that cues presented in one modality/channel typically interact with cues presented in another modality/channel [1]–[3].

For example, attention to visual stimuli is known to influence the perception of speech and other forms of auditory linguistic input [4], [5]. Critical to this study, there is accumulating evidence that cross-modal interactions are highly pronounced in the domain of emotion processing [1], [6]–[8]. The goal of this study was to test the idea that meanings conveyed by emotional prosody systematically influence how listeners visually attend to facial expressions, as inferred from on-line measures of their eye fixation patterns using eye-tracking methodology.

### Emotional processing across sensory modalities

Many studies of emotional processing focus strictly on the visual or auditory modality; this means that far less is known about the processing of multi-modal emotional information, especially when emotional prosody interacts with related social cues. Emotional prosody refers to the melodic and rhythmic components of speech that listeners use to gain insight into a speaker's emotive disposition; a detailed description of the physical (i.e., acoustic) and psycho-perceptual attributes of discrete expressions of emotion through prosody is provided by [9]. Emotional prosody has also been studied independently to establish autonomic events associated with particular expressions [10] and to expose the cognitive and neural architecture involved in prosody encoding and decoding in speech ([11]).

While less studied, the idea that emotional information presented in the auditory channel interacts with visual information is not new. Researchers comparing responses to unimodal versus bimodal emotional stimuli have reported that bimodal (auditory-visual) events facilitate behavioral performance [12], [13] and are associated with increased cerebral activations [14]–[18]. Other studies that have presented auditory and visual stimuli at the same time report that emotional information conveyed in one modality (i.e., by prosody or facial expressions) influences the processing of emotional information in the other modality [1], [8], [19]–[26]. For example, when participants were asked to identify facial expressions that had been morphed between two emotional categories, their decisions were biased in the direction of a simultaneously presented emotional prosody that matched one of the two emotional meanings; conversely, judgments of prosody were biased by a concurrent facial expression in a similar manner [20]. This study suggests that cross-modal influences involving facial and vocal expressions are bi-directional. Various emotional priming paradigms have also yielded evidence of cross-modal interactions, irrespective of what modality is used for presenting the target and/or prime stimulus (visual, auditory, olfactory; [27]–[32]). For instance, Pell and colleagues [23]–[25] have used a cross-modal priming paradigm (Facial Affect Decision Task) where emotionally inflected pseudo-utterances were presented as primes and facial expressions were presented as targets; results of these studies consistently show that participants judge an emotional face more accurately and/or quickly when the target face is preceded by utterances conveying emotionally congruent rather than incongruent prosody. Together, this literature suggests that emotional cues encountered in different sensory modalities are automatically registered for their meaning and that congruent emotional information tends to facilitate behavioral performance in many processing environments (see [33] for a recent discussion).

Emotional congruency effects are generally attributed to the activation of conceptual knowledge about emotion categories shared by different channels of communication [34]–[36], which can be accessed through different sensory modalities. These effects could reflect the ability of congruent events to increase the activation of modality-specific cognitive operations tapped by early electrophysiological components [7], [18], [37], supported by cerebral structures in the superior temporal lobe [17], [38], [39]. Irrespective of the mechanisms involved, much research indicates that when listeners hear emotional prosody they *implicitly* activate underlying emotional meanings even when these features are not the focus of attention [1], [8], [22], [40]–[45]. Given the evidence that auditory and visual cues about emotion interact according to their emotional relationship, and that emotional prosody could be processed outside the focus of attention, it is likely that vocal emotion cues in speech have systematic, implicit effects on visual attention to facial cues in natural social contexts, although this topic has received little attention to date.

### Face processing in the visual search task

The processing of facial expressions has been more widely studied than prosody (see [46]–[48] for reviews). In particular, many researchers have investigated attentional biases to emotional faces using the visual search paradigm [49]–[52], the dot-probe task, [53] and by monitoring on-line eye movements during face processing [54], [55].

There is a general consensus that when participants are presented both emotional and neutral faces, they look longer and more frequently at the emotional faces. However, the relative impact of different emotional faces on visual attention and gaze behavior are less certain. One major claim in the literature is that threat-related expressions (especially anger, and to a lesser extent fear) influence visual processing and attention in a more systematic way than other emotional expressions. For example, visual search tasks show that angry faces (especially schematic ones) are detected more quickly and/or accurately among distractors (either neutral or happy faces) than happy faces among the same expression types [49], [51], [52], [56], [57]. In an eye-tracking study [54], visual arrays of four different schematic faces were presented to participants in three conditions: when the four faces were all identical (either angry, happy, sad, or neutral); when one of the three emotional faces (i.e., angry, happy, or sad) was presented among three neutral faces; and when the four faces were all different. Participants had to decide as quickly as possible whether or not there was a discrepant face in the display. The authors analyzed the probability of first fixations to each face following the onset of the display and the total number of fixations before and after participants made their decision. The results demonstrated that preceding the judgment, the angry faces were fixated significantly less often than neutral faces (experiments 1 and 2). This pattern was interpreted as evidence that angry faces need less time to be identified because they represent important adaptive stimuli related to threat [58].

However, using real faces Williams and colleagues observed that both angry and happy target faces were located more quickly than sad or fearful expressions when presented among neutral distractors using a similar visual search task; this suggested an emotion-specific pattern different from the classical anger-superiority effect [57]. Also, other work [55] has reported shorter saccade latencies towards (real) happy faces when participants were instructed to saccade as quickly as possible to the side where a pre-specified target expression appeared, suggesting that happy faces are identified faster than other emotional expressions depending on task requirements [59], [60]. Taken together, it appears that visual processing biases can occur for both positive and negative-valenced facial expressions depending on task demands, but tend to be most evident for emotions with high adaptive value [61].

Other work using variants of the “visual world” eye-tracking paradigm shows that when participants hear a word, they are more likely to fixate a semantically-related versus semantically-unrelated picture in an array (e.g. [62], [63]). For example, in a passive listening task, Huettig and Altmann [63] instructed participants to scan visual displays accompanied by sentences such as, “*Eventually the man agreed hesitantly, but then he looked at the piano and appreciated that it was beautiful*”. Upon hearing the word *piano*, participants showed a greater tendency to fixate a picture of a semantically related object, such as a trumpet, than on semantically unrelated distractor objects in the display. These results demonstrate that as listeners process auditory cues, visual attention is sensitive to informational congruency between objects in the auditory and visual modalities as inferred from eye movements.

As noted, the effects of emotional prosody on eye movements and attention allocation to facial expressions are still poorly understood. In a very recent undertaking, Paulmann et al. ([64]) employed a visual search paradigm in which participants were explicitly instructed to click on one of five different facial expressions (e.g., “Click on the happy face”), when the instructions were spoken in a congruent or incongruent prosody. Facial expressions were displayed in a circular array around a fixation point, and the frequency and duration of fixations occurring prior to the emotion word (i.e., “happy”) were measured to gauge how prosodic cues influenced gaze *before* participants received explicit information about which face to look at. Results showed that participants made longer and more frequent fixations to facial expressions that were congruent versus incongruent with the emotional prosody of the instruction in the ‘pre-semantic’ time window prior to the emotion word (“Click on the”). This implies that emotional prosody has a rapid and involuntary effect on attention to facial cues during visual search, and in a way that indexes congruent emotional cues between sensory modalities, at least when participants are explicitly instructed to attend to emotional characteristics of the prime-target stimuli [64].

Here, we sought to determine in a more implicit manner whether emotional prosody drives visual search and gaze patterns to congruent facial expressions, in conditions when explicit task requirements did not revolve around emotion (i.e., using an immediate recall task where participants judge whether one emotional face was present in the visual array they had just seen). Similar to [64], we used the eye-tracking technique to sensitively index and track eye fixations to faces but over a longer period of time, allowing insights about the on-line influences of emotional prosody on the way we look at emotional faces and their temporal evolution. We hypothesized that eye movements to faces would be implicitly guided by the meaning of emotional prosody when stimuli are presented concurrently in both modalities, yielding prolonged and more frequent fixations of a facial expression that matched the emotion conveyed by prosody ([64]). Given evidence that certain facial expressions are associated with strong biases in visual processing, we also expected that independent face effects and/or interactions between the emotional expression of faces and the matching status of the emotional prosody would emerge, particularly for facial expressions associated with ‘threat’ such as fear or anger.

## Methods

### Ethics Statement

This research was reviewed and ethically approved by the Faculty of Medicine Institutional Review Board at McGill University (Montréal, Canada). Informed written consent was obtained from each participant prior to entering the study.

### Participants

The participants were 34 native English speakers (17 men/17 women, mean age: 23.6±4.7 years old) who were recruited through campus advertisements. All participants were right-handed and reported normal hearing and normal or corrected-to-normal vision. Before the experiment, each participant completed a questionnaire to establish basic demographic information (age, language abilities) and were formally screened on their levels of anxiety-state and anxiety-trait (STAI, [65]). No participants were excluded on the basis of anxiety scores (mean 34±10 for anxiety-scale; 35±8 for anxiety-state, where a mean of 40 is considered the norm in high-college students, see [65]).

### Apparatus

Stimuli were presented on a View Sonic P95f monitor with Intel Pentium 4 computer. Eye-movements were recorded with an Eye Link II eye tracking system (head mounted video-based; SR Research, Mississauga, Ontario, Canada) connected to an Intel Core2Duo computer (2.79 GHz). The sampling rate of the eye tracker was 500 Hz.

### Stimuli

Materials consisted of emotionally inflected utterances and faces with different emotional expressions. All prosodic and facial stimuli were selected from an existing database of exemplars and have been validated and successfully used in previous work [23]–[25], [66].

#### Auditory stimuli

Given our interest in how prosody (and not semantic information) influences gaze behavior to facial cues, the auditory stimuli presented were emotionally-inflected pseudo-utterances that exploit the phonological and morpho-syntactic properties of English, in the absence of meaningful lexical-semantic cues about emotion (e.g., *Someone migged the pazing*; see [40], [67], [68] for earlier examples). As described by [66], a series of pseudo-utterances was produced by three male and three female speakers (amateur actors) to portray a range of vocal emotions; these utterances were digitally recorded in a sound-attenuated booth, saved as individual audio files, and then perceptually validated by a group of 24 native listeners in a forced-choice emotion recognition task. Based on the data of Pell and colleagues [66], for this study we selected a subset of 64 pseudo-utterances produced by two male and two female speakers, that reliably conveyed fear, anger, happiness, or neutral affect to listeners (16 pseudo-utterances per emotion). The number of items was identical for each speaker and for each emotion. We ensured that the emotional meaning of the prosody for all items was recognized by at least 80% of participants in the validation study [66] and that there were no significant difference in the percentage of target recognition for the selected stimuli across emotional prosody types, *F*(3, 21) = 0.24; *p* = 0.87; see Table 1). As vocal expressions of emotion in speech vary naturally in speech rate and therefore overall duration [9], we edited each pseudo-utterance using Praat software [69] by cutting the stimulus at 1250 ms post-onset of the sentence to avoid any effects of prosody duration on the frequency or duration of eye movements at different time analysis windows (see below for details).

**Table 1:** Major perceptual and physical parameters of the emotional stimuli presented in the experiment.

	Emotion			
Parameters	Fear	Anger	Happiness	Neutrality
Auditory Stimuli (Prosody)				
% Recognition	90±7	88±5	88±6	89±5
Pitch Mean (Hz)	268±41	213±37	179±41	153±37
Pitch Range (Hz)	156±60	186±64	103±37	72±32
Visual Stimuli (Face)				
% Recognition	89±6	87±11	98±3	86±4
Luminance	113.2±13.5	117.0±13.2	115.7±12.7	117.4±9.6
Contrast	42.4±6.4	42.5±5.2	43.4±5.5	44.3±5.5
Skewness	−0.6±0.4	−0.6±0.4	−0.60±0.5	−0.6±0.3
Kurtosis	0.0±0.4	0.11±0.3	0.09±0.5	−0.1±0.4

#### Visual stimuli

All facial expressions selected for the study were color photographs (260×325, 8.5×11 cm) cropped to restrict visual information to facial features. On the basis of the perceptual validation data, we selected 24 emotional faces posed by six actors (three female, three male, different ethnicities). Each actor posed four distinct expressions depicting fear, anger, happiness, and neutrality. All items were correctly recognized by at least 70% of participants in the validation study. Given the role of low-level visual features in guiding eye movements, we controlled major physical parameters of the selected pictures highlighted by previous studies (luminance, contrast for grey and RGB layers, kurtosis, and skewness) using ImageJ software to establish that they did not differ across the emotional sets (see [70]). Physical and perceptual parameters of the selected auditory and facial stimuli used in the experiment are summarized in Table 1.

With these faces, we built a series of visual rectangular arrays composed of four faces posed by the same actor with the four different emotional expressions. The centre of the four pictures was equidistant and localized at 11 cm from the point of fixation. A visual array with four positions, when controlled for spatial arrangement of the faces, resulted in 24 spatially distinct arrays for each of the six actors (144 arrays in total), which were counterbalanced across participants (see below).

### Experimental Design/Procedures

To construct trials, individual auditory stimuli were matched with a visual array, ensuring that the sex of the speaker always matched the sex of the actor in the array, although there was no consistent match between the identity of the speakers and actors who posed the facial expressions. The 64 pseudo-utterances were matched with each of the 24 visual arrays, for a total of 1536 trials; however, to avoid excessive repetition of stimuli within individual subjects and to ensure that the 24 possible spatial arrangements were fully counterbalanced across participants, each participant encountered only one third of the spatial arrangements (n = 512 trials/participant). In addition to the 512 trials in which prosody was presented, 120 visual arrays without concurrent emotional prosody were randomly displayed during the trial sequence to study gaze behavior in the absence of auditory primes (these are referred to as “filler” trials). A total of 632 trials were recorded for each participant.

**Figure 1:**
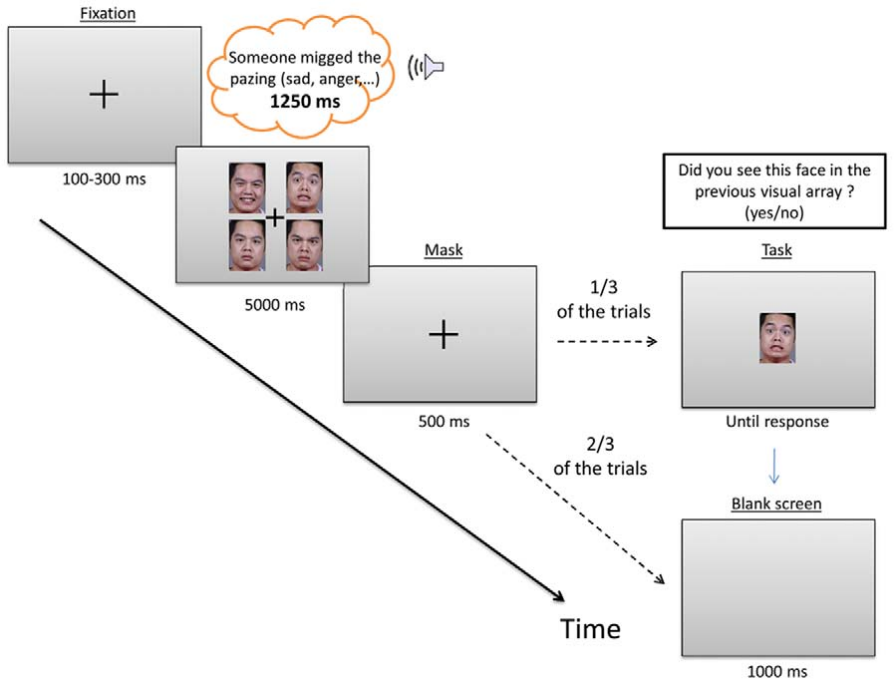
Illustration of the visual array used in this study and of the trial sequence.

Participants were invited to take part in a study of “communication and emotion”; they were seated in a quiet, dimly lit room at a 75 cm distance from the computer screen. Experiment Builder software (SR Research) was used for stimulus presentation. The eye tracker was calibrated at the onset of testing and whenever needed during administration of the experiment. The calibration was accepted if the average error was less than 0.5° in pupil-tracking mode. Each trial began with a centrally-located visual marker that participants were asked to fixate, allowing for drift-correction of the eye-tracker. When the participant's eye was fixated on the circle, the experimenter initiated the trial (see Figure 1). A random delay of 100–300 ms was inserted at the beginning of all trials to prevent anticipatory saccades. Then, the facial array appeared on a grey background for 5000 ms and at the same time a binaural pseudo-utterance was presented over headphones for 1250 ms (the onset of the auditory and facial stimulus was precisely synchronized). Participants were informed that they would often hear a nonsensical sentence when each visual array appeared but that their goal was to simply examine the four faces, because they might be asked to recall which face had been presented in the array for a large number of the trials. Following a mask (500 ms), one third of the trials were followed by a single face which appeared in the center of the computer screen; the participant had to indicate whether he/she had just seen that face (yes/no) by pressing labeled keys on a two-button response box. This procedure ensured that participants were attending to all the faces during each trial. Half of the recall stimuli yielded a “Yes” response (i.e., the single face was one of the previous faces in the array; these trials presented an equal number of exemplars for each of the four emotions of interest) and half of the trials yielded a “No” response (i.e., a facial expression posed by the same actor that conveyed other emotions such as sadness, disgust, surprise, or grimace expressions (see [71] for details). The position of yes and no response buttons was counterbalanced across participants. At the end of each trial, a blank screen appeared for 1000 ms and the next trial was triggered.

Participants completed eleven practice trials before each recording session, which acquainted them with the experimental procedures and features of the stimuli. The experiment lasted approximately 2 hours, administered during two sessions of 60 minutes scheduled two days in a row. After the experiment, the participants were debriefed about the purposes of the study and paid for their participation ($25 CAD).

### Statistical analyses

Data for 31 participants (15 men/16 women; mean age: 23.8±4.8 years old) were considered in all statistical analyses; data for three participants (2 males, 1 female) could not be analyzed due to a technical issue. Behavioral responses were analyzed by running a multivariate ANOVA (MANOVA) with repeated measures of session (first day of testing, second day) and prosody (fear, anger, happy, neutral) on both accuracy and reaction times in the recall task. The multivariate approach to repeated measurements with one measure is similar to a univariate ANOVA and this approach was used to avoid problems with sphericity [72].

For eye-tracking measures, we defined four rectangles that had the same size and location as the four faces in the visual array as target cells. In this way, we were able to measure the frequency and duration of fixations to all target cells which were automatically generated by Data Viewer (SR Research). Whenever participants looked at two different locations in the same target cell in a row, this was counted as two different fixations (with different durations and latencies). Moreover, we computed the latency of the first fixation to one of the faces in the array from presentation onset. Thus, two groups of measures of interest were analyzed: a) the frequency, duration and latency of first saccades being directed to the different faces; b) the number of looks and gaze duration at the different faces in three temporal windows: [0–1250 ms], [1250–2500 ms], [2500–5000 ms]. These temporal windows were chosen to investigate the on-line effects of emotional prosody on gaze behavior when prosodic cues were present and at early and late stages, respectively, following the vocal display.

All measures were entered into separate MANOVAs with repeated measures of matching status of the prosody and the faces fixated (match vs. mismatch) and emotional expression of the face (fear, anger, happiness, neutrality). Given that there were three mismatching and only one matching face in each trial, the frequency or duration of looks to mismatching faces was averaged for each participant prior to analysis. Post-hoc comparisons (Tukey's HSD, *p*<.05) were applied when a significant main or interactive effect was observed. Estimates of effect size were computed as prescribed by [73]. To understand visual scanning patterns when no auditory information was provided to the participants, we also separately analyzed gaze behavior to faces during the 120 filler trials in a similar manner (excluding the *Match* variable which could not be defined for filler trials without prosody).

## Results

### Behavioral performance on the immediate recall task

The overall percentage of faces correctly recalled from the preceding array was high overall (*M* = 90.3%±9.2). Recall accuracy did not differ when the filler trials without prosody were examined separately (*M* = 88.1%±11.8). There was no influence of session or prosody type on recall accuracy, although reaction times were significantly shorter on average for the second (*M* = 1017 ms±195) versus first (*M* = 1271 ms±487) testing session (session main effect: *F*(1, 30) = 6.012; *p* = 0.020).

### Eye gaze measures

The different eye-tracking measures are reported in Tables 2 and 3 for each emotional face expression when accompanied by each type of emotional prosody or without prosody (filler trials).

**Table 2:** Frequency and mean duration (in milliseconds, ms) of fixations measured for the first saccades to a face.

Facial Emotion	Emotional prosody				
	Fear	Anger	Happy	Neutral	None
First Saccade (number/duration, ms)					
Fear	1041/273	1082/261	1048/264	1040/267	1011/266
Anger	913/264	954/264	921/261	978/269	959/265
Happiness	1109/270	1020/274	1075/291	1063/274	884/276
Neutrality	882/247	891/263	903/250	868/254	868/267

**Table 3:** Frequency and mean duration (in milliseconds, ms) of fixations measured in three separate time windows, according to the emotional meaning of the prosody and face.

Facial Emotion	Emotional prosody				
	Fear	Anger	Happy	Neutral	None
Time window = 0–1250 ms (number/duration of looks, ms)					
Fear	2912/251	2988/242	2885/244	2893/245	2613/248
Anger	2525/247	2500/251	2505/245	2488/252	2603/252
Happiness	2876/250	2726/248	2744/254	2843/249	2060/249
Neutrality	2170/242	2216/249	2278/246	2234/247	2338/252
Time window = 1250–2500 ms (number/duration of looks, ms)					
Fear	4092/291	3836/281	3798/285	3817/285	3536/286
Anger	3384/282	3523/291	3301/289	3352/287	3274/299
Happiness	3300/295	3379/394	3630/292	3417/288	2952/287
Neutrality	3161/281	3192/285	3196/285	3314/290	3265/295
Time window = 2500–5000 ms (number/duration of looks, ms)					
Fear	6077/318	5901/313	5903/310	5891/310	5718/308
Anger	5273/315	5639/318	5388/312	5472/315	4829/316
Happiness	5154/316	5086/316	5355/320	5256/320	5131/319
Neutrality	5339/303	5298/305	5314/309	5249/310	5003/312

To understand gaze behavior in the absence of prosody, an one-way MANOVA with repeated measures of *Face* (fear, anger, happiness, neutrality) was performed on the filler trials. The emotional expression of the face significantly influenced the number of first fixations (*F*(3, 28) = 4.924; *p* = 0.007) and the total number of looks at each face (*F*(3, 28) = 20.975; *p*<0.001). Post-hoc analyses revealed a general bias for fearful faces: the number of first fixations was significantly higher to fearful faces than to happy (*p* = 0.008) and neutral (*p* = 0.002) faces, and the total number of looks at fearful faces was greater than for each of the other facial expressions (fear vs. neutral: *p*<0.001; fear vs. anger: *p*<0.001; fear vs. happy: *p*<0.001). The total number of looks at happy faces was also greater than at angry faces overall (*p* = 0.033). There were no significant differences in the *duration* of looks to each facial expression for the filler trials.

When faces were accompanied by prosody, we analyzed two groups of measure. We first interested in the frequency, duration and latency of first saccades being directed to the different faces. Second, we analyzed the number of looks and gaze duration at the different faces in three successive temporal windows to analyze if the pattern of eye movements were changing over time.

### 1. First fixations

#### (a) Number of first fixations

The 2×4 MANOVA with repeated measures of prosody-face *Match* (congruent, incongruent) and emotional facial expression revealed that first fixations were significantly influenced by *Face* (*F*(3, 28) = 7.90; *p* = 0.001). Post-hoc analyses showed that first fixations to happy faces were more frequent than to neutral (*p* = 0.002) and angry faces (*p* = 0.050), and that first fixations to fearful faces were more frequent than to neutral faces (*p* = 0.002). There was no difference in the number of first fixations to happy versus fearful faces or fearful versus angry faces (*p* = 0.255). The extent to which specific facial expressions were fixated initially was not influenced by the matching status of prosody in any manner.

#### (b) Duration of the first fixation

**Figure 2:**
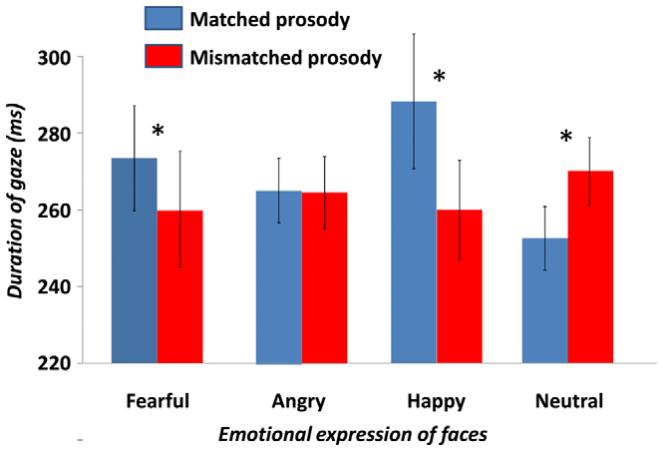
Mean duration of first fixations as a function of facial expression type and the matching status of the prosody (error bars refer to SEM ; *: p<0.05).

The analysis of mean duration of the first fixations revealed a significant effect of *Match* (*F*(1, 30) = 4.201; *p* = 0.049; *r* = 0.350). This effect was qualified by an interaction with the emotional expression of the *Face* (*F*(3, 28) = 3.691; *p* = 0.023; see Figure 2). Follow-up analyses showed that first fixations of happy and fearful faces were significantly longer when accompanied by a matching versus a mismatching prosody (respectively, *p*<0.001 and *p* = 0.020). In contrast, first fixations to neutral faces were longer when they were presented with a *mismatching* (i.e., emotional) prosody than a matching neutral prosody (*p* = 0.044). The duration of first fixations to angry faces did not significantly vary according to *Match*.

#### (c) Latency of the first fixation

Analysis of the latency of the first fixation across trials did not yield any significant effects of *Face* or *Match* (all *p*'s>.050).

### 2. Fixations over time: Time-slice analysis

Given that the prosody could influence gaze behavior differently over time, we delimited three time analysis windows: when prosody was presented concurrently with the faces ([0–1250 ms]; immediately after the prosody was presented [1250–2500 ms]; and more remotely following the prosody [2500–5000 ms]). A *Time window* by *Match* by *Face* (3×2×4) MANOVA was then run on the number of looks and gaze duration, after correcting the number of looks of the third temporal window because it is as twice as long than the two first temporal windows. A three-way interaction of *Time window*×*Match*×*Face* was observed for both measures (number of looks: *F*(6,25) = 4.527; *p* = 0.003; gaze duration: *F*(6,25) = 5.083; *p* = 0.002) allowing us to slice the analysis for each temporal window.

#### (a) [0–1250 ms]

**Figure 3:**
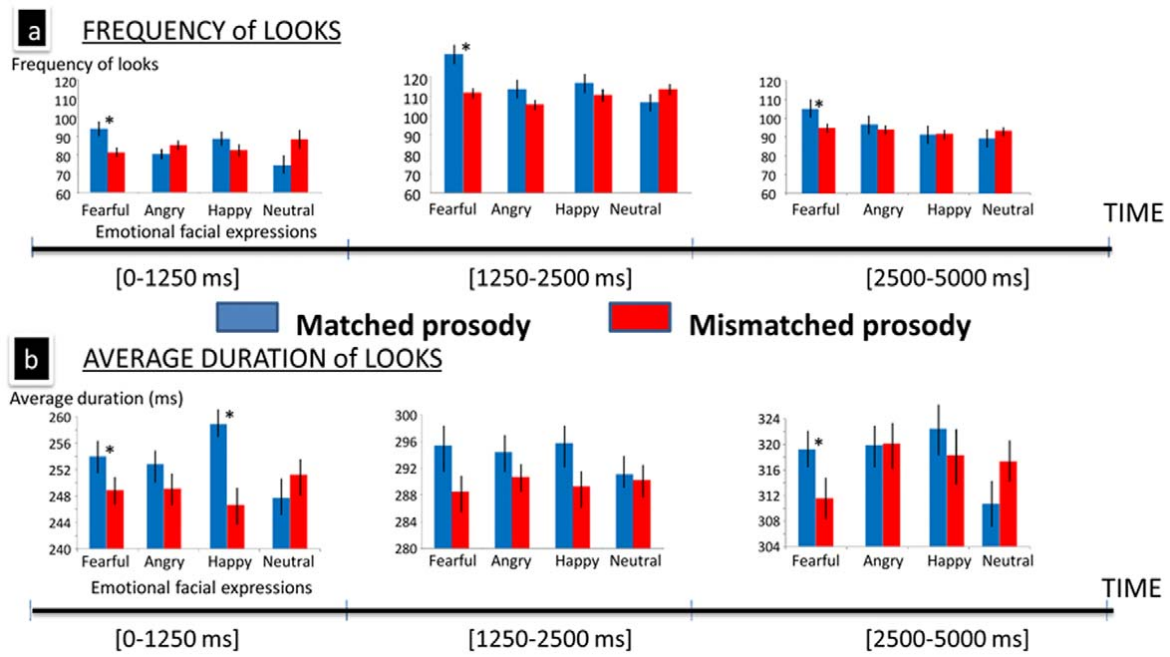
Summary of the time-slice analysis for gaze measures analyzed in three time windows (0–1250 ms, 1250–2500, 2500–5000 ms), illustrating the (a) frequency and (b) mean average duration of looks at faces according to the matching status of the emotional prosody (error bars refer to SEM ; *: p<0.05).

This time window corresponds precisely to the interval when the prosody and facial array were displayed simultaneously in the experiment. For number of looks, we observed an effect of *Face* (*F*(3, 28) = 6.025; *p* = 0.003), which was qualified by a *Match* by *Face* interaction (*F*(3, 28) = 6.948; *p* = 0.001; see Figure 3a). In this early time window, participants looked more often at fearful faces than at angry (*p* = 0.005) and neutral faces (*p*<0.001) overall, and more often at happy than neutral faces (*p*<0.001). Follow-up analyses of the interaction indicated that the influence of matching prosody was only significant for fearful faces, which were looked at more frequently when the prosody matched versus mismatched in the early time window (*p* = 0.002).

When the mean duration of looks in this time window were analyzed, there was a significant main effect of *Match* (*F*(1,30) = 10.586; *p* = 0.003; *r* = 0.511). This effect was qualified by an interaction of *Match* and *Face* (*F*(3,28) = 4.611; *p* = 0.010). Follow-up analyses showed that the mean gaze duration to fearful and happy faces were longer when listeners heard a congruent prosody than an incongruent prosody (significant for happy faces, *p*<0.001; marginally significant for fearful faces, *p* = 0.090).

#### (b) [1250–2500 ms]

This time window allowed us to determine if the effects of matching prosody continue to influence gaze behavior immediately after the auditory stimulus was no longer present. For number of looks, there was a marginally significant effect of *Match* (*F*(1, 30) = 3.619; *p* = 0.067; *r* = 0.328), a significant effect of *Face* (*F*(3, 28) = 7.114; *p* = 0.001), and a significant *Match* by *Face* interaction (*F*(3, 28) = 8.001; *p* = 0.001). Fixations directed at a matching face tended to be more frequent overall than at a mismatching face (117±28 vs. 111±16) and the number of looks at fearful faces was higher overall than at angry, happy and neutral faces (*p*<0.001 for each comparison). The interaction showed that of the four emotional faces, only fear expressions were looked at more frequently when accompanied by a matching versus mismatching prosody (*p*<0.001).

When the mean duration of looks to each face was analyzed for this time window, we observed a significant main effect of *Match* (*F*(1, 30) = 5.187; *p* = 0.029; *r* = 0.384) independent of any main or significant effects involving *Face*. This meant that immediately following the prosodic stimulus, participants looked longer on average at the matching faces than at the mismatching faces (294±41 ms vs. 290±36 ms).

#### (c) [2500–5000 ms]

The final time window allowed us to index the remote effects of a matching/mismatching prosody on fixation patterns during presentation of the visual array. For both the number and duration of looks in this late time window, the respective MANOVA yielded a significant interaction of *Match*×*Face* (number of looks: *F*(3, 28) = 6.605; *p* = 0.002; duration of looks: *F*(3,28) = 3.918; *p* = 0.019). Participants looked more often (*p* = 0.001) and for a longer duration (*p* = 0.046) at fearful faces in trials when the prosody was matching (fearful) versus mismatching. There was no significant effect of *Match* on anger, happy, or neutral facial expressions for either measure in the late time window. It should be noted that a similar time-slice analysis of the filler trials without prosody in the three temporal windows (0–1250 ms, 1250–2500 ms, 2500–5000 ms) yielded similar results in each time window: the number of looks at fearful faces was always more frequent than at the other facial expressions (all *p*'s<0.001).

## Discussion

The main purpose of this study was to test whether emotional speech prosody implicitly guides a listener's attention and eye movements to faces that communicate a congruent emotional meaning, providing on-line evidence that auditory and visual information about emotions are analyzed and integrated during interpersonal events. Behavioral performance in the immediate recall task was high, confirming that our participants focused attention to all faces presented in the visual array as instructed. Although one can argue that participants may have performed well in the recall task without attending carefully to individual faces in the array—for example, by simply applying a strategy of responding ‘yes’ to happy, fear, anger, and neutral faces and ‘no’ to sad, disgust, surprise and grimace faces—our eye-tracking measures show that this was not true, since participants looked at all four faces in the array for 97% of all trials examined. Thus, it is unlikely that a basic lack of attention to events within the facial arrays influenced our results.

Rather, we found that the frequency and duration of fixations to each emotional facial expression were subject to attentional biases, especially for fear, both in conditions when emotional prosody was present as well as absent (see below). Of key interest here, we demonstrated that presenting congruent versus incongruent prosodic information influenced how participants scanned facial expressions and that this cross-modal matching effect evolved over time as the visual array was displayed. The significance of these patterns is discussed in more detail below.

### Scanning facial expressions without prosody: bias for fear

When we analyzed filler trials to understand fixation patterns in the absence of concurrent auditory information, the emotional expression of faces exerted a robust effect on our measures. The first saccades of participants were more often directed towards fearful faces, and across the three temporal windows, participants looked significantly more often at fearful faces than at other facial expressions. This preferential orienting towards fearful faces is coherent with the broader literature on facial expression processing [74], [75], including studies that have used the visual search paradigm [49]–[51], [54] and/or that have analyzed eye-tracking recordings [53], [54], [76]. The fear bias we observed here is probably due to the fact that fearful faces reveal the presence of a threat; from an evolutionary point of view, it has been suggested that the quick detection of threat in the environment provides an important advantage for successful adaptation and may rely on an old subcortical pathway to the amygdala (see [77]).

Although angry faces are also associated with threat, our data show that anger expressions posed by the same actor and presented in the same visual array with fear expressions did not modulate attention and influence eye movements in a similar manner. While fearful faces were fixated more frequently than other expressions, there was no observable bias towards angry faces; in fact, we found that anger faces were looked at less often than happy faces overall. As we carefully controlled how well facial tokens representing each emotion were recognized prior to the study (review Table 1), it is unlikely that our findings reflect differences in the perceptual quality or emotional salience of our facial stimuli across emotion categories. Rather, it is possible that our participants demonstrated an *avoidance* of angry faces, as argued based on eye-tracking results in a related study [78]. In that study, participants passively looked at a visual display of four faces, composed of three neutral faces and one emotional face; in a first experiment, the emotional faces conveyed either happiness or fear, and in a second experiment, either happiness or anger. The authors found that, as early as first saccades, participants tended to avoid looking at the fearful and the angry face; this suggests that they quickly extracted information about the threatening nature of fearful and angry faces, which resulted, rather than overt attention towards, in an avoidance of those threatening faces. The authors interpreted their data as evidence that there is not always an underlying bias to orient towards threat in a rapid and reflexive manner.

This does not clearly explain why fearful faces, which are also related to threat, were not avoided in a similar manner in our experiment. One possible reason that we observed a fear-related (but not anger-related) bias in our data is that fearful and angry faces do not convey the same information about threat; angry faces constitute the threat, whereas fearful faces inform the *presence* of a threat. In the neuroimaging literature it has been shown that the amygdala, a key structure in the processing of emotional information, is more reactive to fearful than angry faces, an effect that has been attributed to the more ambiguous status of fearful faces that are less informative about the location of the threat [79]. This difference in ambiguity and related response tendencies could have played a role in our experimental design, since angry and fearful faces were always displayed at the same time, in contrast to most previous studies. To date, the simultaneous display of anger and fear expressions is rare in eye-tracking studies on the processing of emotional facial expressions; as such, our design may be revealing the effects of competition between these two kinds of threatening faces on visual attention allocation. These ideas merit exploration in the future.

### Scanning facial expressions with prosody: emotion congruency effects over time

The main hypothesis of this study was that the participants would look more often and/or fixate longer at faces conveying the same emotional meaning as prosodic cues presented at the beginning of the visual face array [64]. Many of our gaze measures strongly support this assumption, although as discussed further below, the nature of cross-modal congruency effects often depended on two factors: the time window examined and the discrete emotional properties of the stimuli. When the first fixations of participants were analyzed, we found that they were longer on average when the expression of the face matched the emotional tone of the voice, although this effect was most pronounced for fear and happy expressions. Similarly, the frequency of looks and average duration of fixations measured in the first time window (0–1250 ms) were again sensitive to the shared emotional meanings of vocal and facial cues (main effect of *Match*), but differed significantly only for fearful and happy stimuli. Interestingly, when we analyzed the second time window that immediately followed presentation of the auditory information (1250–2500 ms), we found that the duration of looks at faces that matched the preceding prosody was significantly longer than at mismatching faces *independent* of emotion type.

As noted earlier, many studies have reported emotional congruency effects using behavioral [12], [13], electrophysiological [7], [17], [71] and brain imaging [38], [39] methods, arguing that conceptual knowledge about emotions can be jointly accessed by communicative displays processed in different sensory modalities [35], [36], [80]. In general, our new data support the notion that the cognitive operations involved in processing emotion from speech prosody and from other types of events, such as facial expressions, are tightly intertwined [33]. More specifically, our findings replicate and extend the eye-tracking work of Paulmann et al. [64] who first highlighted that emotional prosody guides eye movements and attention to congruent facial information; using a more explicit paradigm and distinct experimental set-up (involving six emotional faces), they reported longer and more frequent fixations to faces that matched the simultaneous tone of the speaker in a time window where only prosodic cues could guide participants' gaze to a related versus unrelated face.

Here, we further demonstrate that the influence of emotional prosody on gaze behavior persists even when there are no explicit instructions to attend to the emotional content of the voice or face. These data might reflect an implicit need for source identification given the strong link between a face and a voice that characterizes social communication, and evidence from behavioral and neuroimaging studies demonstrating that information from the face and voice are rapidly integrated during person identification [81], [82]. Our results are consistent with investigations indicating that emotional prosody is extracted implicitly irrespective of task instructions or attentional focus [40]–[43]. Thus, while it may be true that a distinct neural network is involved when emotional prosody is processed implicitly versus explicitly [40], it can be said that the impact of emotional prosody on attention allocation to faces is detectable irrespective of whether prosodic information is itself the focus of attention [8], [24], [25].

Our data also emphasize that the influence of emotional prosody on visual behavior fluctuates according to the temporal intervals we defined: in the first time window (during simultaneous cross-modal presentation), the matching effect was mainly driven by fearful and happy voices; in the second time window (after the auditory stimulus was finished), the matching effect was observed evenly for all emotional categories; and in the third (remote) time window, the matching effect was confined to fear. Pending new data which specify the relative time course for recognizing discrete emotions as speech prosody unfolds [83], we argue that the changing effects of emotional prosody on face processing over time reflect differences in how humans ‘prioritize’ or respond to certain emotional cues in the vocal channel, and how vocal cues modulate the activation of emotion knowledge as listeners monitor ongoing prosodic cues in speech to implicitly understand the speaker's meaning.

Specifically, it has been argued that among the basic emotions that are assumed to have discrete expressive properties [84], fear is most salient when expressed in the vocal channel [85]. Recent data also imply that prosodic cues conveying fear are recognized from shorter speech samples than most other emotions, implying that their meanings are activated very quickly [19], [86]. Given the specific relevance of fear signals to humans, combined with the fact that fear is effectively communicated in the vocal channel, it is perhaps unsurprising that prosodic cues conveying fear promoted early, strong cross-modal effects on visual processing of congruent faces during the early time window (see below for further discussion of emotion-specific effects).

In the second time window, the observation that all vocal expressions of emotion as well as neutral expressions influenced gaze toward a congruent face, irrespective of emotion type, merits special commentary; this novel finding could reflect the dynamic and probabilistic nature of vocal emotion recognition [9] and how acoustic fluctuations in speech impact on visual processing. We speculate that after the prosodic information terminated, emotional activations about the prosodic stimulus held in memory had a more stable and robust effect on visual search patterns, influencing how related facial expressions were fixated and yielding a broad congruency effect in this time window. Our new observation that prosody guided saccades to an emotionally-congruent face when auditory information is no longer present suggests that representations of emotional information activated by prosody are maintained in memory for a certain period of time, perhaps approximating 1000–1250 ms [45], [87]. It has been proposed elsewhere that these representations are in fact supramodal (see [80] for a discussion).

Data for our third time window imply that representations for fear activated by prosodic information last longer in memory than for other emotions, as this was the only emotion to produce a matching effect in our remote time window (an interval ranging from 1250 to 3750 ms following completion of the auditory stimulus). Results for our third time window are in line with the importance of keeping meaningful information, such as events related to fear and threat, in mind longer as has been demonstrated by some research [45], [88]–[91].

Although emotion-specific effects are routinely described in other studies of emotional prosody [92]–[95] and emotional facial expressions [96], [97], some of the emotion-specific patterns in our data were unexpected in certain respects. While our measures supply clear evidence that fearful, and to a lesser extent happy faces, were looked at longer in many conditions when the emotional prosody matched the face, neutral faces were looked at longer when the prosody *mismatched* the face (i.e., when the prosody conveyed emotion). There were relatively few emotion-specific effects related to anger in our study. Note that these differential effects emerged despite the fact that fearful, angry and neutral faces all had very similar levels of target recognition prior to the study. In light of recent work focusing on the behavioral and neuro-cognitive effects of listening to angry voices [1], [19], it is surprising that angry voices did not consistently influence how participants allocate visual attention to faces, yielding a similar matching effect in conditions where this was selectively observed for fear and happy. Based on recent findings, it seems that anger processing may be differentially sensitive to cross-modal congruency effects during stimulus processing; for example, in an electrophysiological investigation that presented prosody-face pairs, Paulmann and Pell [71] observed that the P200 component was larger in response to congruent fearful versus congruent angry trials. Also, Park et al. [98] found different patterns of activation in the middle temporal gyrus in response to bimodal fearful versus angry stimuli composed of both emotionally congruent sentence and face. Further research will be needed to understand the emotion-specific effects revealed in our study, particularly for anger.

The fact that we observed a reverse congruency effect for neutral stimuli (i.e., participants looked longer at neutral faces when the prosody was emotional than when it was neutral) is noteworthy; this may showcase a general tendency for emotional prosody to capture and direct the visual attention of listeners when compared to neutral prosody. Overt emotional cues in the voice may have aroused participants to deploy more attentional resources to facial analysis (including neutral faces); indeed, the broad ability of emotional information to arouse organisms, as dispositions to action [99], by increases of peripheral activity like heart rate and skin conductance is widely recognized. Our results imply that emotional prosody could have a general impact on visual search behavior as well. As summarized by Juslin and Lauka [9], vocal expressions of emotion are associated with distinct acoustic configurations (e.g., changes in fundamental frequency, intensity and duration) which include differences in vocal arousal or intensity (perceived loudness and vocal effort). Since neutral utterances are typically produced with very low intensity/little physiological arousal when compared to most emotional utterances [66], this could explain why neutral prosody had minimal effects on gaze patterns to emotional faces. On the other hand, since our emotional expressions were pre-rated for their intensity and did not differ for anger and fear stimuli (review Table 1), it is unlikely that any differences in perceived intensity or arousal explain the discrepancies we sometimes observed between anger and fear. Further experiments with different emotional expressions will help to disentangle these emotion-specific effects; for example, electrophysiological investigations could add valuable data on the temporal course of the effects observed in this study, with a specific interest in early components such as N2pc which is thought to represent attentional selection and is elicited in visual search paradigms (see [100]).

### Conclusion

Expressions of the face and of the voice are often embedded together as core features of human social interactions [101], [102]. Cross-modal interactions between the voice and face channels therefore have important social implications as, for example, hearing a voice helps to identify a person [82], [83], to make perceptual decisions [103] and various inferences can be made from one modality to the other [104]. In light of evidence that auditory information helps to efficiently deploy visual attention towards relevant information such as emotional stimuli [19], audiovisual integration has an unquestionable adaptive value for humans.

Our study represents one of the first accounts to show that emotional prosody guides how we gaze at faces. These results merit attention because the cross-modal effects we observed occurred even when prosody was not relevant to task goals, and the influence of emotional prosody on visual behavior persisted after auditory information was no longer present. Our study opens up new possibilities as to how eye-tracking recordings may be used by other researchers to gather new information on the nature of cross-modal and multimodal emotion processing. For example, it would be interesting in a follow-up study to display angry (or fearful) faces within a visual array of neutral or happy faces (like in [78]) to understand some of the patterns observed in our present study. On the basis of studies showing that the particular emotional expression of a face dictates how it is visually scanned (see [105]), it would also be interesting to investigate whether congruent and incongruent emotional prosody influences fixation patterns to different regions of a single emotional face. Without doubt, our findings show that emotional prosody shapes our visual representation of the social environment by guiding visual exploration in systematic ways, reinforcing the importance of vocal information in how we apprehend the world and how we represent it in the brain.
